# Supplementary material for: Quantifying the association between ethnicity and COVID-19 mortality: a national cohort study protocol
Source: BMJ Open. 2021 Apr 7;11(4):e045286. doi: 10.1136/bmjopen-2020-045286 (PMC8029035; doi:10.1136/bmjopen-2020-045286)
Supplement: Author's manuscript [file bmjopen-2020-045286.draft_revisions.pdf]

BMJ Open is committed to open peer review. As part of this commitment we make the peer review history of every article we publish publicly available.

When an article is published we post the peer reviewers' comments and the authors' responses online. We also post the versions of the paper that were used during peer review. These are the versions that the peer review comments apply to.

The versions of the paper that follow are the versions that were submitted during the peer review process. They are not the versions of record or the final published versions. They should not be cited or distributed as the published version of this manuscript.

BMJ Open is an open access journal and the full, final, typeset and author-corrected version of record of the manuscript is available on our site with no access controls, subscription charges or pay-per-view fees (http://bmjopen.bmj.com).

If you have any questions on BMJ Open's open peer review process please email info.bmjopen@bmj.com

BMJ Open

Quantifying the association between ethnicity and COVID-19 mortality: A national cohort study protocol

Journal:	*BMJ Open*
Manuscript ID	bmjopen-2020-045286
Article Type:	Protocol
Date Submitted by the Author:	30-Sep-2020
Complete List of Authors:	Dambha-Miller, Hajira; University of Southampton Faculty of Medicine, Department of Primary Care; University of Cambridge, MRC Epidemiology Unit<br>Saatci, Defne; University of Oxford, Nuffield Department of Primary Care Health Science<br>Clift, Ashley; University of Oxford, Nuffield Department of Primary Care Health Sciences<br>Zaccardi, Francesco; University of Leicester, Diabetes Research Centre<br>Coupland, Carol; University of Nottingham, Division of Primary Care<br>Locufier , Patrick; PPI Representative<br>Davies, Firoza; PPI Representative<br>Khunti, Kamlesh; University of Leicester, Department of Health Sciences<br>Griffin, Simon; The Primary Care Unit, Institute of Public Health<br>Hippisley-Cox, Julia; University of Oxford, Nuffield Department of Primary Care Sciences
Keywords:	COVID-19, EPIDEMIOLOGY, PUBLIC HEALTH

SCHOLARONE™
Manuscripts

I, the Submitting Author has the right to grant and does grant on behalf of all authors of the Work (as defined in the below author licence), an exclusive licence and/or a non-exclusive licence for contributions from authors who are: i) UK Crown employees; ii) where BMJ has agreed a CC-BY licence shall apply, and/or iii) in accordance with the terms applicable for US Federal Government officers or employees acting as part of their official duties; on a worldwide, perpetual, irrevocable, royalty-free basis to BMJ Publishing Group Ltd ("BMJ") its licensees and where the relevant Journal is co-owned by BMJ to the co-owners of the Journal, to publish the Work in this journal and any other BMJ products and to exploit all rights, as set out in our licence.

The Submitting Author accepts and understands that any supply made under these terms is made by BMJ to the Submitting Author unless you are acting as an employee on behalf of your employer or a postgraduate student of an affiliated institution which is paying any applicable article publishing charge ("APC") for Open Access articles. Where the Submitting Author wishes to make the Work available on an Open Access basis (and intends to pay the relevant APC), the terms of reuse of such Open Access shall be governed by a Creative Commons licence – details of these licences and which Creative Commons licence will apply to this Work are set out in our licence referred to above.

Other than as permitted in any relevant BMJ Author's Self Archiving Policies, I confirm this Work has not been accepted for publication elsewhere, is not being considered for publication elsewhere and does not duplicate material already published. I confirm all authors consent to publication of this Work and authorise the granting of this licence.

For peer review only

Quantifying the association between ethnicity and COVID-19 mortality: A national cohort study protocol

Hajira Dambha-Miller[1], Defne Saatci[2], Ashley Clift[2], Francesco Zaccardi[3], Carol Coupland[4], Patrick Locufier[5], Firoza Davies[5], Kamlesh Khunti[3], Simon Griffin[6], Julia Hippisley-Cox[2]

1. Division of Primary Care and Population Health, University of Southampton
2. Nuffield Department of Primary Care Health Sciences, University of Oxford
3. Leicester Diabetes Centre, Leicester General Hospital, University of Leicester, Leicester, UK
4. Division of Primary Care, School of Medicine, University of Nottingham
5. PPI representative
6. Department of Public Health and Primary Care, University of Cambridge

Correspondence: Dr Hajira Dambha-Miller, Primary Care and Population Health, University of Southampton, Southampton, SO16 5ST, Email: H.Dambha-Miller@doctors.org.uk

For peer review only

Abstract

Introduction

Recent evidence suggests that ethnic minority groups are disproportionately at increased risk of hospitalisation and death from SARS-CoV-2 infection. Population-based evidence on potential explanatory and mediating factors across minority groups and within sub-groups is lacking. This study aims to quantify the association between ethnicity and the risk of hospitalisation and mortality due to COVID-19.

Methods and analysis

A prospective cohort study of adults registered across a representative and anonymised national primary care database (QResearch) that includes data on 10 million people in the UK. Sociodemographic, deprivation, clinical and domicile characteristics will be summarised and compared across ethnic subgroups (categorised as per 2011 census). Cox models will be used to calculate hazard ratios for hospitalisation and COVID-19 mortality associated with ethnic group. Potential confounding and explanatory factors (such as demographic, socioeconomic and clinical) will be adjusted for within regression models. The percentage contribution of distinct risk factor classes to the excess risks seen in ethnic groups/sub-groups will be calculated.

Ethics and dissemination

The study is undergone ethics review in accordance with the QResearch agreement (reference OX102). Findings will be disseminated through peer-reviewed manuscripts, presentations at scientific meetings and conferences with national and international stakeholders.

Strengths and limitations

- The Q-Research database includes a large sample that represents 18% of the UK population
- As the data is based on primary care clinical entries, recording of exposures and national linkage to outcomes but this may vary in completeness.
- Total COVID-19 cases in the cohort will be under-estimated as national systematic testing is still being established in the UK

For peer review only

Introduction

Recent reports and early observational data suggest that the prevalence of COVID-19, hospitalisation and deaths are disproportionately higher amongst ethnic minority populations. [1–3] Potential speculative mechanisms include genetic variations, co-morbidities, cultural or behavioural patterns, occupational factors, social constructs and inequalities. In the UK, people with ethnic minority backgrounds constitute 16% of the population but the Intensive Care National Audit and Research Centre report that over 30% of people admitted to intensive care with COVID-19 were from these backgrounds.[4,5] Furthermore, 64% of 119 NHS staff who died from COVID-19 as of 30th April 2020 were from ethnic minority backgrounds.[6] A rapid review on the subject led by Khunti et al. suggests that there are also variations in mortality within broad ethnic minority groups.[3] Most national and international data on COVID-19 are aggregated and have not reported outcome by ethnic group sufficiently to permit examination of within-group differences.

To date, a limited number of population-based cohort studies regarding COVID-19 have been undertaken worldwide [7-10]. The UK is unique in the availability of high quality, large (multi-million) databases that have linkage across varying information sources. These are being used, for example, to develop risk prediction models for adverse COVID-19 outcomes in the general population [11], and to undertake large scale epidemiological evaluations of COVID-19 risk factors and drug safety. In the UK, two large cohorts have reported to date on the association between ethnicity and COVID-19 related mortality outcomes: the OpenSAFELY Collaborative of 23 million UK records and the NHS England cohort study of over 61 million people. The OpenSAFELY consortium reported that people with Black or Asian backgrounds have a higher risk of death from COVID-19 compared to people who are White (HR 1.5 and 1.4, respectively) [7]. Data from NHS England also showed a greater mortality risk for Asian (HR 1.3) and Black backgrounds (HR 1.7) [12]. Further analysis of this data, carried out by the Office of National Statistics (ONS), showed that the mortality risk in Black men and women was 1.9 times higher than in White individuals [13]. Contradictory to a previous study in a smaller, single-state US health system database, [14], both studies identify a persisting excess risk of COVID-19 specific mortality after accounting for known risk factors, including age, sex, deprivation and co-morbidities such as obesity, diabetes and cardiovascular disease. The causes of these excess risks are poorly understood, incompletely quantified and may vary within and between ethnic minority groups.

Previous studies have been limited by the lack of consideration for additional potential mediators, such as specific medications, rarer comorbidities, or social differences such as household structure. A recent population-based study, for example, identified a significant interaction between ethnicity and angiotensin enzyme inhibitors (ACEi)/angiotensin receptor blockers (ARBs) for COVID-19 disease [15]. Several smaller studies have also suggested a role for glucose-lowering medications in the ethnicity-dependent variations observed in COVID-19 outcomes [16,17]. Single-centre studies and case-series are providing additional insights into the potential interaction between ethnicity and haematological disorders, such as sickle cell disease, in relation to COVID-19 [18,19]. Population-based studies with adequate number of people from different ethnic groups that consider these wider variables are necessary to evaluate the explanatory factors surrounding this risk. Accordingly, in this study we aim to quantify ethnic group-specific risk of SARS-CoV-2 infection, COVID-19 mortality and COVID-19 hospital admission, and examine the effect of non-modifiable (e.g. age and sex) and explanatory factors on the associations between ethnicity and mortality/hospitalisation risk.

For peer review only

For peer review only - http://bmjopen.bmj.com/site/about/guidelines.xhtml

Methods

Study Design

A retrospective cohort study from electronic GP records linked at the individual level to hospital admissions, SARS-CoV-2 testing and death registry databases.

Data sources

We will utilise the QResearch national primary care database that includes 1205 general practices with 10 million records. The sample will represent 18% of the UK population. QResearch holds data on a heterogeneous and representative ethnic minority population, with comprehensive information on a range of clinical and socioeconomic factors. Additional databases may be included as these become available.

Study period

We will include a period from the date of the first case of COVID-19 in the UK (24th January 2020) until death or the latest date for which complete data are available at the time of analysis.

Inclusion criteria

All adults aged over 18 years registered with a GP practice during the study period and for at least 12 months prior to inclusion. The GP practice registration needs to be linked to a relevant clinical system (i.e. EMIS). The index date will be the first recorded instance of the outcomes of interest.

Primary Outcome

The primary outcome will be a COVID-19 related mortality. COVID-19 mortality will be defined as either confirmed or suspected COVID-19 on a death certificate, or death from any cause in a patient with a confirmed positive RT-PCR viral SARS-CoV-2 test in the immediately preceding 28 days.

Secondary Outcomes

Confirmed SARS-CoV-2 infection, hospitalisation due to COVID-19 and ITU admission (which will also include patients receiving mechanical ventilation). Hospitalisation will be defined as hospitalisation with confirmed or suspected COVID-19 (as per ICD-10 codes), or new hospitalisation in a patient with a positive SARS-CoV-2 test in the immediately preceding 28 days.

Ethnicity definition

A self-reported ethnic group is recorded in medical records. QResearch has ethnicity records in 80% of all patients. Read codes for ethnicity will be collapsed into the nine categories of the last UK census. Ethnicity will be considered in two ways; first in the 9 high-level categories: White, Indian, Bangladeshi, Pakistani, other Asian, Black African, Black Caribbean, Chinese, 'Other' and then, if numbers allow, further subdivided into the standard 17 categories used by ONS.

Explanatory Variables

We will extract data (where available) corresponding to the following potential exposure and confounding variables. This list provided below is indicative and correct as of 29h August 2020, but will be kept under review as new information arises in the literature:

Demographic variables

- Ethnicity (9 categories, and numbers permitting, sub-division into 17 ONS groups)
- Sex
- Age (continuous variable)
- Geographical region

For peer review only - http://bmjopen.bmj.com/site/about/guidelines.xhtml

For peer review only

Concurrent medication

- Glucose-lowering medications (metformin, sulfonylureas, thiazolindione, SGLT-2 inhibitors, DPP-4 inhibitors, GLP-1R agonists) and insulin therapy
- Hormone therapy (in women)

Clinical/blood test assessments

- Body mass index
- Blood pressure (systolic and diastolic)
- HbA1c
- Total cholesterol and LDL
- Liver function tests, including alanine aminotransferase
- eGFR

Comorbidities

- Sickle Cell Status
  - Sickle Trait
  - Sickle Cell Disease

- Diabetes (type 1 and type 2 separately)
  - Diabetes duration

- Chronic kidney disease
  - CKD stage 3
  - CKD stage 4
  - CKD stage 5 (including those with requiring dialysis or ever receiving a transplant)

- Cancer
  - Active or past haematological or solid cancer

- Transplant (solid organ or bone marrow)

Key confounding variables

Demographic variables

- Quintile of Townsend deprivation score. This is an area-level quintile score based on the patients' postcode. Originally developed by Townsend, it includes unemployment (as a percentage of those aged 16 and over who are economically active); non-car ownership (as a percentage of all households); non-home ownership (as a percentage of all households) and household overcrowding. These variables are measured for a given area of approximately 120 households, via the 2011 census, and combined to give a "Townsend score" for that area. A higher Townsend score implies a greater level of deprivation.
- Residence type
  - Lives in a care home (nursing home or residential care)
  - Homelessness
  - Lives in own home
- Household characteristics
  - Age of household members
  - Number of household inhabitants

Lifestyle factors

- Smoking status coded as non-smoker, ex-smoker, light [<10 cigarettes/day], moderate [10-19], heavy [20+])

Comorbidities

For peer review only - http://bmjopen.bmj.com/site/about/guidelines.xhtml

- Cardiovascular disease
- Hypertension
- Chronic respiratory disease
- Liver cirrhosis
- Depression
- Dementia
- Other chronic neurological diseases
- Severe mental health illness
- Learning Disability

Concurrent medication

- Lipid-lowering medications
- Antihypertensive medications

All predictor variables will be based on the latest coded information recorded in the GP record prior to entry to the cohort.

Sample size calculation

As of the 9th Aug 2020, there had been 41,686 COVID-19 deaths in England out of a total population of 56 million. Assuming a 0.074% mortality rate in the general population, to detect a conservative estimate of a hazard ratio of 1.2 for COVID-19 mortality in the ethnic minority population that represents 18.6% of the study population (based on recent QResearch studies) with 90% power and a significance level of 0.01, we would need a sample size of 3,995,350 individuals with 2,957 COVID-19 deaths. As recent studies of COVID-19 using QResearch have included cohorts of over 8 million adults with over 5,000 COVID-19 deaths, even with 40% missing data, our study is sufficiently powered.

Statistical methods

Participant characteristics will be summarised by ethnicity, age, sex, co-morbidities and outcome with appropriate summary statistics. We will describe SARS-CoV-2 infection, COVID-19 hospitalisation and COVID-19 mortality rates in the general population and stratify this by ethnicity. Plots of survival curves stratified by ethnic group will be generated using the Kaplan-Meier method and compared with the log-rank test. We will use Cox proportional hazards models to estimate the unadjusted and adjusted hazard ratios for associations between ethnicity and the outcomes of interest. Subsequently, we will seek to quantify the contribution of risk factor groups to any observed increased mortality or hospitalisation risks in people of non-white ethnicity (mediation analyses). For all analyses, we will conduct complete case analyses, and separately evaluate the models using multiply imputed data (as a sensitivity analysis). Under the 'missing at random assumption', we will use multiple imputation with chained equations to generate 5 imputed datasets, where values for ethnicity, body mass index, clinical variables (such as blood pressure, cholesterol), Townsend deprivation quintile and smoking status are imputed. Imputation models will include all exposure and outcome variables; statistical models will be developed on each of the 5 imputed datasets and the estimates pooled using Rubin's rules. Data will be analysed using STATA (version 16). Our study will be conducted and findings reported in line with the STROBE [20] and RECORD guidelines [21] for observational studies using routinely collected health data.

Patient and public involvement

Patient representatives have been involved in the development of the study aims and design. We sought feedback from study inception and design from patients and the public within the Centre for BME Health, Leicester. We additionally have two public representatives as part of the study team whose contributions have been acknowledged in the authorship list of this manuscript. They will continue to be involved in all stages of study with additional public and patient views sought for dissemination strategies.

For peer review only - http://bmjopen.bmj.com/site/about/guidelines.xhtml

Ethics and dissemination

The project has been independently peer-reviewed and received ethics approval by the QResearch Scientific board (reference OX102). Findings will be disseminated through peer-reviewed manuscripts, presentations at scientific meetings and conferences with national and international stakeholders.

Discussion

Our findings aim to provide rapid evidence on patterns of COVID-19 and associated mortality across and within ethnic groups in the UK. This study will be one of the largest COVID-19 cohorts in the UK, with a representative population across both rural and urban areas. Our cohort size will enable us to detect national-level variations within ethnic minority subgroups across a large geographical area. Thus, to some extent, overcome selection bias limitations of previous cohort studies. Our findings have the potential to inform targeted mitigation public health strategies and could alter clinical thresholds for at-risk patients presenting with the infection.

As we are using routinely recorded clinical data from primary care records rather than prospectively collecting the data, we anticipate that our study will be subject to limitations. First, the precision and completeness of routine NHS patient records will vary although previous work from QResearch suggests that key variables have high levels of accuracy and completeness. Second, our sample is likely to under-estimate the total number of people in the population with COVID-19 due to the lack of systematic national testing programmes which are still being rolled out, and also due to false-negative test results. There is also some evidence that testing rates are lower amongst ethnic minority groups. Our confirmed SARS-CoV-2 infection cases will additionally over-represent people with more severe disease who died or were hospitalised. During the earlier part of the pandemic, testing in the UK was limited to the hospital setting and people with COVID-19 who were not admitted or recovered (who may be from ethnic minority groups) are less likely to be captured in the data. Further, the admission criteria for ITU varied during the course of the pandemic with particular characteristics such as older age resulting in a lower chance of admission at a time when high admission rates were anticipated, which may introduce a selection bias.

Funding

This work is funded by an MRC grant (MR/V027778/1). SJG is supported by an MRC Epidemiology Unit programme: MC_UU_12015/4. The University of Cambridge has received salary support in respect of SJG from the NHS in the East of England through the Clinical Academic Reserve. HDM is a National Institute for Health Research funded Academic Clinical Lecturer. JHC receives support from the NHS and National Institute for Health Research and various research councils. KK and FZ are supported by the National Institute for Health Research (NIHR) Applied Research Collaboration East Midlands (ARC EM) and the NIHR Leicester Biomedical Research Centre (BRC). The views and opinions expressed by authors in this publication are those of the authors and do not necessarily reflect those of the UK National Institute for Health Research (NIHR) or the Department of Health and Social Care.

Competing Interests

JHC is founder and director of QResearch database which is not-for-profit organisation with EMIS (leading commercial supplier of IT for 55% of general practices in the UK). JHC is co-owner of ClinRisk Ltd and was a paid director until June 2019. ClinRisk Ltd develops open and closed source software to ensure the reliable and updatable implementation of clinical risk equations within clinical computer systems to help improve patient care outside the submitted work. KK is national lead for NIHR ARC ethnicity and diversity, Director for the University of Leicester Centre for BME Health, Trustee of the South Asian Health Foundation and member of the Independent SAGE. The authors declare that no support from any organisation and no financial relationships have influenced the submitted work.

For peer review only - http://bmjopen.bmj.com/site/about/guidelines.xhtml

Contributor statement

HDM drafted the protocol and all authors contributed towards revising it.

References

1 Pareek M, Bangash M, Pareek N et al. (2020) Ethnicity and COVID-19: an urgent public health research priority. *Lancet*. 395(10234): 1421-1422

2 Laurencin CT, McClinton A. (2020) The COVID-19 Pandemic: a Call to Action to Identify and Address Racial and Ethnic Disparities. *Journal of Racial and Ethnic Health Disparities.* 7, 398–402

3 Razaq A., Harrison D., et al. (2020) BAME COVID-19 DEATHS - What do we know? Rapid Data & Evidence Review CEBM. https://www.cebm.net/covid-19/bame-covid-19-deaths-what-do-we-know-rapid-data-evidence-review/ (accessed 10th August 2020).

4 Intensive Care National Audit & Research Centre – Reports. https://www.icnarc.org/Our-Audit/Audits/Cmp/Reports (accessed 10th August 2020).

5 Ethnic minority deaths and Covid-19, The King's Fund. https://www.kingsfund.org.uk/blog/2020/04/ethnic-minority-deaths-covid-19 (accessed 4 May 2020).

6 Rimmer A. (2020) Covid-19: Two thirds of healthcare workers who have died were from ethnic minorities. BMJ. 369:m1621.

7 Williamson, E.J., Walker, A.J., Bhaskaran, K. *et al.* (2020) OpenSAFELY: factors associated with COVID-19 death in 17 million patients. *Nature.* https://doi.org/10.1038/s41586-020-2521-4

8 Emilsson V, Gudmundsson EF, Aspelund T, et al. (2020) ACE2 levels are altered in comorbidities linked to severe outcome in COVID-19. Preprint. *MedRxiv*. doi:10.1101/2020.06.04.20122044

9 Kim J, Kim DW, Kim KI, et al. (2020) Compliance of Antihypertensive Medication and Risk of Coronavirus Disease 2019: a Cohort Study Using Big Data from the Korean National Health Insurance Service. *J Korean Med Sci*. 35(25):e232.

10 Radon K, Saathoff E, Pritsch M, et al. (2020) Protocol of a population-based prospective COVID-19 cohort study Munich, Germany (KoCo19). *BMC Public Health*. 20(1):1036.

11 Hippisley-Cox, J., Clift A., Coupland C., et al. (2020). "Protocol for the development and evaluation of a tool for predicting risk of short-term adverse outcomes due to COVID-19 in the general UK population." *medRxiv:* 2020.2006.2028.20141986.

12 Barron E, Bakhai C., Kar P., et al., (2020) Type 1 and Type 2 diabetes and COVID-19 related mortality in England: a whole population study. https://www.england.nhs.uk/wp-content/uploads/2020/05/valabhji-COVID-19-and-Diabetes-Paper-1.pdf

13 Office of National Statistics. 2020. https://www.ons.gov.uk/peoplepopulationandcommunity/birthsdeathsandmarriages/deaths/articles/coronavirusrelateddeathsbyethnicgroupenglandandwales/2march2020to10april2020

14 Price-Haywood EG, Burton J, Fort D et al., (2020) Hospitalization and Mortality among Black Patients and White Patients with Covid-19. *N Engl J Med*. 382(26):2534-2543.

15 Hippisley-Cox, J, Young, D, Couplan C, et al., Risk of severe COVID-19 disease with ACE inhibitors and angiotensin receptor blockers: cohort study including 8.3 million people. Heart. 2020 Jul 31. pii: heartjnl-2020-317393. doi: 10.1136/heartjnl-2020-317393.

For peer review only

For peer review only - http://bmjopen.bmj.com/site/about/guidelines.xhtml

16 Zhu L, She ZG, Cheng X, et al. Association of Blood Glucose Control and Outcomes in Patients with COVID-19 and Pre-existing Type 2 Diabetes. *Cell Metab*. 2020;31(6):1068-1077.e3. doi:10.1016/j.cmet.2020.04.021

17 Scheen AJ, Marre M, Thivolet C. Prognostic factors in patients with diabetes hospitalized for COVID-19: Findings from the CORONADO study and other recent reports [published online ahead of print, 2020 May 21]. *Diabetes Metab*. 2020;S1262-3636(20)30085-9. doi:10.1016/j.diabet.2020.05.008

18 Panepinto, J.A., et al., Coronavirus Disease among Persons with Sickle Cell Disease, United States, March 20-May 21, 2020. Emerg Infect Dis, 2020. 26(10)

19 Arlet, J.B., et al., Prognosis of patients with sickle cell disease and COVID-19: a French experience. Lancet Haematol, 2020.

20 Elm E, Altman D, Egger M et al. (2007) Strengthening the reporting of observational studies in epidemiology (STROBE) statement: guidelines for reporting observational studies *BMJ* 335 :806

21 Langan S, Schmidt SAJ, Wing K, et al. (2018) The reporting of studies conducted using observational routinely collected health data statement for pharmacoepidemiology (RECORD-PE) *BMJ.* 363 :k3532

For peer review only

STROBE 2007 (v4) checklist of items to be included in reports of observational studies in epidemiology*
Checklist for cohort, case-control, and cross-sectional studies (combined)

Section/Topic	Item #	Recommendation	Reported on page #
**Title and abstract**	1	(*a*) Indicate the study's design with a commonly used term in the title or the abstract	2-3
		(*b*) Provide in the abstract an informative and balanced summary of what was done and what was found	2
**Introduction**			
Background/rationale	2	Explain the scientific background and rationale for the investigation being reported	3
Objectives	3	State specific objectives, including any pre-specified hypotheses	3
**Methods**			
Study design	4	Present key elements of study design early in the paper	4
Setting	5	Describe the setting, locations, and relevant dates, including periods of recruitment, exposure, follow-up, and data collection	4-5
Participants	6	(*a*) *Cohort study*—Give the eligibility criteria, and the sources and methods of selection of participants. Describe methods of follow-up<br>*Case-control study*—Give the eligibility criteria, and the sources and methods of case ascertainment and control selection. Give the rationale for the choice of cases and controls<br>*Cross-sectional study*—Give the eligibility criteria, and the sources and methods of selection of participants	5-6
		(*b*) *Cohort study*—For matched studies, give matching criteria and number of exposed and unexposed<br>*Case-control study*—For matched studies, give matching criteria and the number of controls per case	n/a
Variables	7	Clearly define all outcomes, exposures, predictors, potential confounders, and effect modifiers. Give diagnostic criteria, if applicable	6-7
Data sources/ measurement	8*	For each variable of interest, give sources of data and details of methods of assessment (measurement). Describe comparability of assessment methods if there is more than one group	7
Bias	9	Describe any efforts to address potential sources of bias	7
Study size	10	Explain how the study size was arrived at	6
Quantitative variables	11	Explain how quantitative variables were handled in the analyses. If applicable, describe which groupings were chosen and why	6
Statistical methods	12	(*a*) Describe all statistical methods, including those used to control for confounding	6
		(*b*) Describe any methods used to examine subgroups and interactions	n/a
		(*c*) Explain how missing data were addressed	n/a
		(*d*) *Cohort study*—If applicable, explain how loss to follow-up was addressed<br>*Case-control study*—If applicable, explain how matching of cases and controls was addressed	n/a

For peer review only

		Cross-sectional study—If applicable, describe analytical methods taking account of sampling strategy	
		(*e*) Describe any sensitivity analyses	8
**Results**			
Participants	13*	(a) Report numbers of individuals at each stage of study—eg numbers potentially eligible, examined for eligibility, confirmed eligible, included in the study, completing follow-up, and analysed	8-9
		(b) Give reasons for non-participation at each stage	n/a
		(c) Consider use of a flow diagram	n/a
Descriptive data	14*	(a) Give characteristics of study participants (eg demographic, clinical, social) and information on exposures and potential confounders	8-9
		(b) Indicate number of participants with missing data for each variable of interest	n/a
		(c) *Cohort study*—Summarise follow-up time (eg, average and total amount)	n/a
Outcome data	15*	*Cohort study*—Report numbers of outcome events or summary measures over time	n/a
		Case-control study—Report numbers in each exposure category, or summary measures of exposure	n/a
		Cross-sectional study—Report numbers of outcome events or summary measures	n/a
Main results	16	(*a*) Give unadjusted estimates and, if applicable, confounder-adjusted estimates and their precision (eg, 95% confidence interval). Make clear which confounders were adjusted for and why they were included	n/a
		(*b*) Report category boundaries when continuous variables were categorized	n/a
		(*c*) If relevant, consider translating estimates of relative risk into absolute risk for a meaningful time period	n/a
Other analyses	17	Report other analyses done—eg analyses of subgroups and interactions, and sensitivity analyses	7
**Discussion**			
Key results	18	Summarise key results with reference to study objectives	n/a
Limitations	19	Discuss limitations of the study, taking into account sources of potential bias or imprecision. Discuss both direction and magnitude of any potential bias	7
Interpretation	20	Give a cautious overall interpretation of results considering objectives, limitations, multiplicity of analyses, results from similar studies, and other relevant evidence	n/a protocol study
Generalisability	21	Discuss the generalisability (external validity) of the study results	7
**Other information**			
Funding	22	Give the source of funding and the role of the funders for the present study and, if applicable, for the original study on which the present article is based	7

*Give information separately for cases and controls in case-control studies and, if applicable, for exposed and unexposed groups in cohort and cross-sectional studies.

Note: An Explanation and Elaboration article discusses each checklist item and gives methodological background and published examples of transparent reporting. The STROBE checklist is best used in conjunction with this article (freely available on the Web sites of PLoS Medicine at http://www.plosmedicine.org/, Annals of Internal Medicine at http://www.annals.org/, and Epidemiology at http://www.epidem.com/). Information on the STROBE Initiative is available at www.strobe-statement.org.

For peer review only

BMJ Open

Quantifying the association between ethnicity and COVID-19 mortality: A national cohort study protocol

Journal:	*BMJ Open*
Manuscript ID	bmjopen-2020-045286.R1
Article Type:	Protocol
Date Submitted by the Author:	28-Jan-2021
Complete List of Authors:	Dambha-Miller, Hajira; University of Southampton Faculty of Medicine, Department of Primary Care; University of Cambridge, MRC Epidemiology Unit<br>Tan, Pui San; University of Oxford, Nuffield Department of Primary Care Health Sciences<br>Saatci, Defne; University of Oxford, Nuffield Department of Primary Care Health Science<br>Clift, Ashley; University of Oxford, Nuffield Department of Primary Care Health Sciences<br>Zaccardi, Francesco; University of Leicester, Diabetes Research Centre<br>Coupland, Carol; University of Nottingham, Division of Primary Care<br>Locufier , Patrick; PPI Representative<br>Davies, Firoza; PPI Representative<br>Khunti, Kamlesh; University of Leicester, Department of Health Sciences<br>Griffin, Simon; The Primary Care Unit, Institute of Public Health<br>Hippisley-Cox, Julia; University of Oxford, Nuffield Department of Primary Care Sciences
**Primary Subject Heading**:	Epidemiology
Secondary Subject Heading:	Infectious diseases
Keywords:	COVID-19, EPIDEMIOLOGY, PUBLIC HEALTH

SCHOLARONE™
Manuscripts

I, the Submitting Author has the right to grant and does grant on behalf of all authors of the Work (as defined in the below author licence), an exclusive licence and/or a non-exclusive licence for contributions from authors who are: i) UK Crown employees; ii) where BMJ has agreed a CC-BY licence shall apply, and/or iii) in accordance with the terms applicable for US Federal Government officers or employees acting as part of their official duties; on a worldwide, perpetual, irrevocable, royalty-free basis to BMJ Publishing Group Ltd ("BMJ") its licensees and where the relevant Journal is co-owned by BMJ to the co-owners of the Journal, to publish the Work in this journal and any other BMJ products and to exploit all rights, as set out in our licence.

The Submitting Author accepts and understands that any supply made under these terms is made by BMJ to the Submitting Author unless you are acting as an employee on behalf of your employer or a postgraduate student of an affiliated institution which is paying any applicable article publishing charge ("APC") for Open Access articles. Where the Submitting Author wishes to make the Work available on an Open Access basis (and intends to pay the relevant APC), the terms of reuse of such Open Access shall be governed by a Creative Commons licence – details of these licences and which Creative Commons licence will apply to this Work are set out in our licence referred to above.

Other than as permitted in any relevant BMJ Author's Self Archiving Policies, I confirm this Work has not been accepted for publication elsewhere, is not being considered for publication elsewhere and does not duplicate material already published. I confirm all authors consent to publication of this Work and authorise the granting of this licence.

For peer review only

Quantifying the association between ethnicity and COVID-19 mortality: A national cohort study protocol

Hajira Dambha-Miller[1], Pui Tan[2,] Defne Saatci[2], Ashley Clift[2], Francesco Zaccardi[3], Carol Coupland[4], Patrick Locufier[5], Firoza Davies[5], Kamlesh Khunti[3], Simon Griffin[6] and Julia Hippisley-Cox[2]

1. Division of Primary Care and Population Health, University of Southampton
2. Nuffield Department of Primary Care Health Sciences, University of Oxford
3. Leicester Diabetes Centre, Leicester General Hospital, University of Leicester, Leicester, UK
4. Division of Primary Care, School of Medicine, University of Nottingham
5. PPI representative
6. Department of Public Health and Primary Care, University of Cambridge

Correspondence: Dr Hajira Dambha-Miller, Primary Care and Population Health, University of Southampton, Southampton, SO16 5ST, Email: hajiradambha@doctors.org.uk

For peer review only

For peer review only - http://bmjopen.bmj.com/site/about/guidelines.xhtml

Abstract

Introduction

Recent evidence suggests that ethnic minority groups are disproportionately at increased risk of hospitalisation and death from SARS-CoV-2 infection. Population-based evidence on potential explanatory factors across minority groups and within sub-groups is lacking. This study aims to quantify the association between ethnicity and the risk of hospitalisation and mortality due to COVID-19.

Methods and analysis

A retrospective cohort study of adults registered across a representative and anonymised national primary care database (QResearch) that includes data on 10 million people in the UK. Sociodemographic, deprivation, clinical and domicile characteristics will be summarised and compared across ethnic subgroups (categorised as per 2011 census). Cox models will be used to calculate hazard ratios for hospitalisation and COVID-19 mortality associated with ethnic group. Potential confounding and explanatory factors (such as demographic, socioeconomic and clinical) will be adjusted for within regression models. The percentage contribution of distinct risk factor classes to the excess risks seen in ethnic groups/sub-groups will be calculated.

Ethics and dissemination

The study is undergone ethics review in accordance with the QResearch agreement (reference OX102). Findings will be disseminated through peer-reviewed manuscripts, presentations at scientific meetings and conferences with national and international stakeholders.

Strengths and limitations

- The Q-Research database includes a large sample that represents 18% of the UK population
- As the data is based on primary care clinical entries, recording of exposures and national linkage to outcomes but this may vary in completeness.
- Total COVID-19 cases in the cohort will be under-estimated as national systematic testing is still being established in the UK

For peer review only - http://bmjopen.bmj.com/site/about/guidelines.xhtml

Introduction

Recent reports and early observational data suggest that the prevalence of COVID-19, hospitalisation and deaths are disproportionately higher amongst ethnic minority populations [1–3]. Potential speculative mechanisms include genetic variations, co-morbidities, cultural or behavioural patterns, occupational factors, social constructs and inequalities. In the UK, people with ethnic minority backgrounds constitute 16% of the population but the Intensive Care National Audit and Research Centre report that over 30% of people admitted to intensive care with COVID-19 were from these backgrounds [4,5]. Furthermore, 64% of 119 NHS staff who died from COVID-19 as of 30th April 2020 were from ethnic minority backgrounds [6]. A rapid review on the subject led by Khunti et al. suggests that there are also variations in mortality within broad ethnic minority groups.[3] Most national and international data on COVID-19 are aggregated and have not reported outcome by ethnic group sufficiently to permit examination of within-group differences.

At the time of submitting this manuscript, a limited number of population-based cohort studies regarding COVID-19 have been undertaken worldwide. In the USA, a study within the largest healthcare system, the Veterans Health Administration, reports excess mortality amongst ethnic minorities.[7] In the UK, the availability of high quality, large (multi-million) databases that have linkage across varying information sources are starting to examine this association further. Large primary care database have been used, for example, to develop risk prediction models for adverse COVID-19 outcomes in the general population , and to undertake large scale epidemiological evaluations of COVID-19 risk factors and drug safety [8]. In the UK, two large cohorts have reported to date on the association between ethnicity and COVID-19 related mortality outcomes: the OpenSAFELY Collaborative of 23 million UK records and the NHS England cohort study of over 61 million people. The OpenSAFELY consortium reported that people with Black or Asian backgrounds have a higher risk of death from COVID-19 compared to people who are White (HR 1.5 and 1.4, respectively) [9]. Data from NHS England also showed a greater mortality risk for Asian (HR 1.3) and Black backgrounds (HR 1.7). Further analysis of this data, carried out by the Office of National Statistics (ONS), showed that the mortality risk in Black men and women was 1.9 times higher than in White individuals. Contradictory to a previous study in a smaller, single-state US health system database,, both studies identify a persisting excess risk of COVID-19 specific mortality after accounting for known risk factors, including age, sex, deprivation and co-morbidities such as obesity, diabetes and cardiovascular disease. The causes of these excess risks are poorly understood, incompletely quantified and may vary within and between ethnic minority groups.

Previous studies have been limited by the lack of consideration of additional potential explanatory variables, such as specific medications, rarer comorbidities [10], social differences such as household structure or vaccination status.[9] A recent population-based study, for example, identified a significant interaction between ethnicity and angiotensin enzyme inhibitors (ACEi)/angiotensin receptor blockers (ARBs) for COVID-19 disease [11]. Several smaller studies have also suggested a role for glucose-lowering medications in the ethnicity-dependent variations observed in COVID-19 outcomes [12,13]. Single-centre studies and case-series are providing additional insights into the potential interaction between ethnicity and haematological disorders, such as sickle cell disease [10], in relation to COVID-19. Population-based studies with adequate number of people from different ethnic groups that consider these wider variables are necessary to evaluate the explanatory factors surrounding this risk. Moreover, earlier studies have been limited to England, larger cohorts that extend to the wider UK population are needed for validation and to allow generalisability of findings. Accordingly, in this study we aim to quantify ethnic group-specific risk of SARS-CoV-2 infection, COVID-19 mortality and COVID-19 hospital admission, and examine the effect of non-modifiable (e.g. age and sex) and explanatory factors on the associations between ethnicity and mortality/hospitalisation risk.

For peer review only - http://bmjopen.bmj.com/site/about/guidelines.xhtml

Methods

Study Design

A retrospective cohort study from electronic GP records linked at the individual level to hospital admissions, SARS-CoV-2 testing and death registry databases.

Data sources

We will utilise the QResearch national primary care database that includes 1205 general practices with 10 million records. The sample represents 18% of the UK population. QResearch holds data on a heterogeneous and representative ethnic minority population, with comprehensive information on a range of clinical and socioeconomic factors. Further detail on the population included within the Q-Research database and representative is updated regularly on the database website. [14] Additional databases may be included as these become available.

Study period

We will include a period from the date of the first case of COVID-19 in the UK (24th January 2020) until death or the latest date for which complete data are available at the time of analysis.

Inclusion criteria

All adults aged over 18 years registered with a GP practice during the study period and for at least 12 months prior to inclusion. The GP practice registration needs to be linked to a relevant clinical system (i.e. EMIS). The index date will be the first recorded instance of the outcomes of interest.

Primary Outcome

The primary outcome will be a COVID-19 related mortality as recorded in the primary care records and linked to ONS data. Details on the extent of linkage available within the database has been reported previously and can be found in full on the Q-Research website.[15] COVID-19 related mortality will be defined as the presence of U07.1 or U07.2 on death certificates or a death from any cause in a patient with a confirmed positive SARS-CoV-2 test in the immediately preceding 28 days.

Secondary Outcomes

COVID-19 hospitalisation was defined as hospitalisation with confirmed or suspected COVID-19 (as per ICD-10 codes), or new hospitalisation in a patient with a positive SARS-CoV-2 test in the immediately preceding 14 days.

Ethnicity definition

A self-reported ethnic group is recorded in medical records. QResearch has ethnicity records in 80% of all patients. Ethnicity will be considered in two ways; first in the 9 high-level categories: White, Indian, Bangladeshi, Pakistani, other Asian, Black African, Black Caribbean, Chinese, ‘Other’ and then, if numbers allow, further subdivided into the standard 17 categories used by ONS and include the following; English/Welsh/Scottish/Northern Irish/British, Irish, Gypsy or Irish Traveller, Any other White background, White and Black Caribbean, White and Black African, White and Asian, Any other Mixed/Multiple ethnic backgrounds, Indian, Pakistani, Bangladeshi, Chinese, Any other Asian background, African, Caribbean, Any other Black/African/Caribbean background, Arab or any other ethnic group.

For peer review only - http://bmjopen.bmj.com/site/about/guidelines.xhtml

For peer review only

Explanatory Variables

We will extract data (where available) corresponding to the following potential exposure and confounding variables. This list provided below is indicative and correct as of 29h August 2020, but will be kept under review as new information arises in the literature:

Demographic variables
- Ethnicity (9 categories, and numbers permitting, sub-division into 17 ONS groups)
- Sex
- Age (continuous variable)
- Geographical region

Concurrent medication
- Glucose-lowering medications (metformin, sulfonylureas, thiazolindione, SGLT-2 inhibitors, DPP-4 inhibitors, GLP-1R agonists) and insulin therapy
- Hormone therapy (in women)

Clinical/blood test assessments
- Body mass index
- Blood pressure (systolic and diastolic)
- HbA1c
- Total cholesterol and LDL
- Liver function tests, including alanine aminotransferase
- eGFR

Comorbidities
- Sickle Cell Status [10]
  - Sickle Trait
  - Sickle Cell Disease

- Diabetes (type 1 and type 2 separately) [13,16]
  - Diabetes duration

- Chronic kidney disease
  - CKD stage 3
  - CKD stage 4
  - CKD stage 5 (including those with requiring dialysis or ever receiving a transplant)

- Cancer
  - Active or past haematological or solid cancer

- Transplant (solid organ or bone marrow)
- Vaccination status

Key confounding variables

Demographic variables
- Quintile of Townsend deprivation score. This is an area-level quintile score based on the patients' postcode. Originally developed by Townsend, it includes unemployment (as a percentage of those aged 16 and over who are economically active); non-car ownership (as a percentage of all households); non-home ownership (as a percentage of all households) and household overcrowding. These variables are measured for a given area of approximately 120 households, via the 2011 census, and combined to give a "Townsend score" for that area. A higher Townsend score implies a greater level of deprivation.
- Residence type

For peer review only - http://bmjopen.bmj.com/site/about/guidelines.xhtml

- Lives in a care home (nursing home or residential care)
- Homelessness
- Lives in own home

- Household characteristics
  - Age of household members
  - Number of household inhabitants

Lifestyle factors

- Smoking status coded as non-smoker, ex-smoker, light [<10 cigarettes/day], moderate [10-19], heavy [20+])

Comorbidities

- Cardiovascular disease
- Hypertension
- Chronic respiratory disease
- Liver cirrhosis
- Depression
- Dementia
- Other chronic neurological diseases
- Severe mental health illness
- Learning Disability

Concurrent medication

- Lipid-lowering medications
- Antihypertensive medications

All predictor variables will be based on the latest coded information recorded in the GP record prior to entry to the cohort.

Sample size calculation

As of the 9th Aug 2020, there had been 41,686 COVID-19 deaths in England out of a total population of 56 million. Assuming a 0.074% mortality rate in the general population, to detect a conservative estimate of a hazard ratio of 1.2 for COVID-19 mortality in the ethnic minority population that represents 18.6% of the study population (based on recent QResearch studies) with 90% power and a significance level of 0.01, we would need a sample size of 3,995,350 individuals with 2,957 COVID-19 deaths. As recent studies of COVID-19 using QResearch have included cohorts of over 8 million adults with over 5,000 COVID-19 deaths, even with 40% missing data, our study is sufficiently powered.

Statistical methods

Participant characteristics will be summarised by ethnicity, age, sex, co-morbidities and outcome with appropriate summary statistics. We will describe SARS-CoV-2 infection, COVID-19 hospitalisation and COVID-19 mortality rates in the general population and stratify this by ethnicity. Plots of survival curves stratified by ethnic group will be generated using the Kaplan-Meier method and compared with the log-rank test. We will use Cox proportional hazards models to estimate the unadjusted and adjusted hazard ratios for associations between ethnicity and the outcomes of interest. When proportional hazards assumptions are not met, we will consider using more flexible models which do not assume proportionality of hazards e.g. flexible parametric survival models. Subsequently, we will seek to quantify the contribution of risk factor groups to any observed increased mortality or hospitalisation risks in people of non-white ethnicity. For all analyses, we will conduct complete case analyses, and then separately impute for missing values e.g. ethnic group, BMI, deprivation quintile and smoking status using five imputations incorporating all model outcomes and predictors with model coefficients and standard errors pooled in accordance with Rubin's rules. Data will be analysed using STATA (version 16). Our study will be conducted and findings reported in line with the STROBE [17] and RECORD guidelines for observational studies using routinely collected health data[18].

For peer review only - http://bmjopen.bmj.com/site/about/guidelines.xhtml

Patient and public involvement

Patient representatives have been involved in the development of the study aims and design. We sought feedback from study inception and design from patients and the public within the Centre for BME Health, Leicester. We additionally have two public representatives as part of the study team whose contributions have been acknowledged in the authorship list of this manuscript. They will continue to be involved in all stages of study with additional public and patient views sought for dissemination strategies.

Ethics and dissemination

The project has been independently peer-reviewed and received ethics approval by the QResearch Scientific board (reference OX102). Findings will be disseminated through peer-reviewed manuscripts, presentations at scientific meetings and conferences with national and international stakeholders.

Data sharing agreement

Data are available by direct requests to the Q-Research database. We are working towards making our code publicly available.

Discussion

Our findings aim to provide rapid evidence on patterns of COVID-19 and associated mortality across and within ethnic groups in the UK. This study will be one of the largest COVID-19 cohorts in the UK, with a representative population across both rural and urban areas. Our cohort size will enable us to detect national-level variations within ethnic minority subgroups across a large geographical area. Thus, to some extent, overcome selection bias limitations of previous cohort studies. Our findings have the potential to inform targeted mitigation public health strategies and could alter clinical thresholds for at-risk patients presenting with the infection.

As we are using routinely recorded clinical data from primary care records rather than prospectively collecting the data, we anticipate that our study will be subject to limitations. First, the precision and completeness of routine NHS patient records will vary although previous work from QResearch suggests that key variables have high levels of accuracy and completeness. Second, our sample is likely to under-estimate the total number of people in the population with COVID-19 due to the lack of systematic national testing programmes which are still being rolled out, and also due to false-negative test results. There is also some evidence that testing rates are lower amongst ethnic minority groups. Our confirmed SARS-CoV-2 infection cases will additionally over-represent people with more severe disease who died or were hospitalised. During the earlier part of the pandemic, testing in the UK was limited to the hospital setting and people with COVID-19 who were not admitted or recovered (who may be from ethnic minority groups) are less likely to be captured in the data. Further, the admission criteria for ITU varied during the course of the pandemic with particular characteristics such as older age resulting in a lower chance of admission at a time when high admission rates were anticipated, which may introduce a selection bias.

Funding

This work is funded by an MRC grant (MR/V027778/1). SJG is supported by an MRC Epidemiology Unit programme: MC_UU_12015/4. The University of Cambridge has received salary support in respect of SJG from the NHS in the East of England through the Clinical Academic Reserve. HDM is a National

For peer review only - http://bmjopen.bmj.com/site/about/guidelines.xhtml

Institute for Health Research funded Academic Clinical Lecturer. JHC receives support from the NHS and National Institute for Health Research and various research councils. KK and FZ are supported by the National Institute for Health Research (NIHR) Applied Research Collaboration East Midlands (ARC EM) and the NIHR Leicester Biomedical Research Centre (BRC). The views and opinions expressed by authors in this publication are those of the authors and do not necessarily reflect those of the UK National Institute for Health Research (NIHR) or the Department of Health and Social Care.

Competing Interests

JHC is founder and director of QResearch database which is not-for-profit organisation with EMIS (leading commercial supplier of IT for 55% of general practices in the UK). JHC is co-owner of ClinRisk Ltd and was a paid director until June 2019. ClinRisk Ltd develops open and closed source software to ensure the reliable and updatable implementation of clinical risk equations within clinical computer systems to help improve patient care outside the submitted work. KK is national lead for NIHR ARC ethnicity and diversity, Director for the University of Leicester Centre for BME Health, Trustee of the South Asian Health Foundation and member of the Independent SAGE. The authors declare that no support from any organisation and no financial relationships have influenced the submitted work.

Contributor statement

HDM led the study conceptualisation, contributed to study design, wrote the first draft and revised the protocol, PT critically revised the manuscript, DS critically revised the manuscript, AC critically revised the manuscript. FZ critically revised the manuscript and contributed to the statistical methods, CC critically revised the manuscript and contributed to the statistical methods, PL critically revised the manuscript as a PPI contributor, FD critically revised the manuscript as a PPI contributor, KK contributed to study conceptualisation, study design and critically revised the manuscript, SG contributed to study conceptualisation, study design and critically revised the manuscript, JHC contributed to study conceptualisation, study design and critically revised the manuscript. The corresponding author attests that all listed authors meet authorship criteria and that no others meeting the criteria have been omitted. HDM is the guarantor.

.

References

1 Pareek M, Bangash M, Pareek N et al. 2020. Ethnicity and COVID-19: an urgent public health research priority - The Lancet. doi:OI:https://doi.org/10.1016/S0140-6736(20)30922-3

2 Laurencin CT, McClinton A. The COVID-19 Pandemic: a Call to Action to Identify and Address Racial and Ethnic Disparities. *Journal of Racial and Ethnic Health Disparities* 2020;:1. doi:10.1007/s40615-020-00756-0

3 BAME COVID-19 DEATHS - What do we know? Rapid Data & Evidence Review - CEBM. https://www.cebm.net/covid-19/bame-covid-19-deaths-what-do-we-know-rapid-data-evidence-review/ (accessed 6 May 2020).

4 ICNARC – Reports. https://www.icnarc.org/Our-Audit/Audits/Cmp/Reports (accessed 4 May 2020).

5 Ethnic minority deaths and Covid-19, The King's Fund. https://www.kingsfund.org.uk/blog/2020/04/ethnic-minority-deaths-covid-19 (accessed 4 May 2020).

6 Rimmer A. Covid-19: Two thirds of healthcare workers who have died were from ethnic minorities. *BMJ* 2020;**369**:m1621. doi:10.1136/bmj.m1621

7 Rentsch ID CT, Kidwai-Khan F, Tate JP, *et al.* Patterns of COVID-19 testing and mortality by race and ethnicity among United States veterans: A nationwide cohort study. doi:10.1371/journal.pmed.1003379

8 Clift AK, Coupland CAC, Keogh RH, *et al.* Living risk prediction algorithm (QCOVID) for risk of hospital admission and mortality from coronavirus 19 in adults: national derivation and validation cohort study. *The BMJ* 2020;**371**. doi:10.1136/bmj.m3731

9 OpenSAFELY Collaborative T, Mathur R, Rentsch CT, *et al.* Ethnic differences in COVID-19 infection, hospitalisation, and mortality: an OpenSAFELY analysis of 17 million adults in

For peer review only - http://bmjopen.bmj.com/site/about/guidelines.xhtml

England. *medRxiv* 2020;:2020.09.22.20198754. doi:10.1101/2020.09.22.20198754
10 Arlet JB, de Luna G, Khimoud D, *et al.* Prognosis of patients with sickle cell disease and COVID-19: a French experience. The Lancet Haematology. 2020;**7**:e632–4. doi:10.1016/S2352-3026(20)30204-0
11 Hippisley-Cox J, Young D, Coupland C, *et al.* Risk of severe COVID-19 disease with ACE inhibitors and angiotensin receptor blockers: cohort study including 8.3 million people. *Heart (British Cardiac Society)* 2020;**106**:1503–11. doi:10.1136/heartjnl-2020-317393
12 Zhu L, She ZG, Cheng X, *et al.* Association of Blood Glucose Control and Outcomes in Patients with COVID-19 and Pre-existing Type 2 Diabetes. *Cell Metabolism* 2020;**31**:1068-1077.e3. doi:10.1016/j.cmet.2020.04.021
13 Scheen AJ, Marre M, Thivolet C. Prognostic factors in patients with diabetes hospitalized for COVID-19: Findings from the CORONADO study and other recent reports. Diabetes and Metabolism. 2020;**46**:265–71. doi:10.1016/j.diabet.2020.05.008
14 Summary of GP population characteristics - QResearch. https://www.qresearch.org/data/summary-of-gp-population-characteristics/#h-H2_0 (accessed 24 Jan 2021).
15 Summary Analysis of Linked Mortality Data - QResearch. https://www.qresearch.org/data/summary-analysis-of-linked-mortality-data/ (accessed 24 Jan 2021).
16 Barron E, Bakhai C, Kar P, *et al.* Associations of type 1 and type 2 diabetes with COVID-19-related mortality in England: a whole-population study. *The Lancet Diabetes and Endocrinology* 2020;**8**:813–22. doi:10.1016/S2213-8587(20)30272-2
17 Von Elm E, Altman DG, Egger M, *et al.* The Strengthening the Reporting of Observational Studies in Epidemiology (STROBE) statement: Guidelines for reporting observational studies. Annals of Internal Medicine. 2007;**147**:573–7. doi:10.7326/0003-4819-147-8-200710160-00010
18 Benchimol EI, Smeeth L, Guttmann A, *et al.* The REporting of studies Conducted using Observational Routinely-collected health Data (RECORD) Statement. *PLoS Medicine* 2015;**12**. doi:10.1371/journal.pmed.1001885

For peer review only

For peer review only - http://bmjopen.bmj.com/site/about/guidelines.xhtml

STROBE 2007 (v4) checklist of items to be included in reports of observational studies in epidemiology*
Checklist for cohort, case-control, and cross-sectional studies (combined)

Section/Topic	Item #	Recommendation	Reported on page #
**Title and abstract**	1	(*a*) Indicate the study's design with a commonly used term in the title or the abstract	2-3
		(*b*) Provide in the abstract an informative and balanced summary of what was done and what was found	2
**Introduction**			
Background/rationale	2	Explain the scientific background and rationale for the investigation being reported	3
Objectives	3	State specific objectives, including any pre-specified hypotheses	3
**Methods**			
Study design	4	Present key elements of study design early in the paper	4
Setting	5	Describe the setting, locations, and relevant dates, including periods of recruitment, exposure, follow-up, and data collection	4-5
Participants	6	(*a*) *Cohort study*—Give the eligibility criteria, and the sources and methods of selection of participants. Describe methods of follow-up<br>*Case-control study*—Give the eligibility criteria, and the sources and methods of case ascertainment and control selection. Give the rationale for the choice of cases and controls<br>*Cross-sectional study*—Give the eligibility criteria, and the sources and methods of selection of participants	5-6
		(*b*) *Cohort study*—For matched studies, give matching criteria and number of exposed and unexposed<br>*Case-control study*—For matched studies, give matching criteria and the number of controls per case	n/a
Variables	7	Clearly define all outcomes, exposures, predictors, potential confounders, and effect modifiers. Give diagnostic criteria, if applicable	6-7
Data sources/ measurement	8*	For each variable of interest, give sources of data and details of methods of assessment (measurement). Describe comparability of assessment methods if there is more than one group	7
Bias	9	Describe any efforts to address potential sources of bias	7
Study size	10	Explain how the study size was arrived at	6
Quantitative variables	11	Explain how quantitative variables were handled in the analyses. If applicable, describe which groupings were chosen and why	6
Statistical methods	12	(*a*) Describe all statistical methods, including those used to control for confounding	6
		(*b*) Describe any methods used to examine subgroups and interactions	n/a
		(*c*) Explain how missing data were addressed	n/a
		(*d*) *Cohort study*—If applicable, explain how loss to follow-up was addressed<br>*Case-control study*—If applicable, explain how matching of cases and controls was addressed	n/a

For peer review only

		Cross-sectional study—If applicable, describe analytical methods taking account of sampling strategy	
		(*e*) Describe any sensitivity analyses	8
**Results**			
Participants	13*	(a) Report numbers of individuals at each stage of study—eg numbers potentially eligible, examined for eligibility, confirmed eligible, included in the study, completing follow-up, and analysed	8-9
		(b) Give reasons for non-participation at each stage	n/a
		(c) Consider use of a flow diagram	n/a
Descriptive data	14*	(a) Give characteristics of study participants (eg demographic, clinical, social) and information on exposures and potential confounders	8-9
		(b) Indicate number of participants with missing data for each variable of interest	n/a
		(c) *Cohort study*—Summarise follow-up time (eg, average and total amount)	n/a
Outcome data	15*	*Cohort study*—Report numbers of outcome events or summary measures over time	n/a
		Case-control study—Report numbers in each exposure category, or summary measures of exposure	n/a
		Cross-sectional study—Report numbers of outcome events or summary measures	n/a
Main results	16	(*a*) Give unadjusted estimates and, if applicable, confounder-adjusted estimates and their precision (eg, 95% confidence interval). Make clear which confounders were adjusted for and why they were included	n/a
		(*b*) Report category boundaries when continuous variables were categorized	n/a
		(*c*) If relevant, consider translating estimates of relative risk into absolute risk for a meaningful time period	n/a
Other analyses	17	Report other analyses done—eg analyses of subgroups and interactions, and sensitivity analyses	7
**Discussion**			
Key results	18	Summarise key results with reference to study objectives	n/a
Limitations	19	Discuss limitations of the study, taking into account sources of potential bias or imprecision. Discuss both direction and magnitude of any potential bias	7
Interpretation	20	Give a cautious overall interpretation of results considering objectives, limitations, multiplicity of analyses, results from similar studies, and other relevant evidence	n/a protocol study
Generalisability	21	Discuss the generalisability (external validity) of the study results	7
**Other information**			
Funding	22	Give the source of funding and the role of the funders for the present study and, if applicable, for the original study on which the present article is based	7

*Give information separately for cases and controls in case-control studies and, if applicable, for exposed and unexposed groups in cohort and cross-sectional studies.

Note: An Explanation and Elaboration article discusses each checklist item and gives methodological background and published examples of transparent reporting. The STROBE checklist is best used in conjunction with this article (freely available on the Web sites of PLoS Medicine at http://www.plosmedicine.org/, Annals of Internal Medicine at http://www.annals.org/, and Epidemiology at http://www.epidem.com/). Information on the STROBE Initiative is available at www.strobe-statement.org.

For peer review only

Quantifying the association between ethnicity and COVID-19 mortality: A national cohort study protocol

Journal:	*BMJ Open*
Manuscript ID	bmjopen-2020-045286.R2
Article Type:	Protocol
Date Submitted by the Author:	07-Mar-2021
Complete List of Authors:	Dambha-Miller, Hajira; University of Southampton Faculty of Medicine, Department of Primary Care; University of Cambridge, MRC Epidemiology Unit<br>Tan, Pui San; University of Oxford, Nuffield Department of Primary Care Health Sciences<br>Saatci, Defne; University of Oxford, Nuffield Department of Primary Care Health Science<br>Clift, Ashley; University of Oxford, Nuffield Department of Primary Care Health Sciences<br>Zaccardi, Francesco; University of Leicester, Diabetes Research Centre<br>Coupland, Carol; University of Nottingham, Division of Primary Care<br>Locufier , Patrick; PPI Representative<br>Davies, Firoza; PPI Representative<br>Khunti, Kamlesh; University of Leicester, Department of Health Sciences<br>Griffin, Simon; The Primary Care Unit, Institute of Public Health<br>Hippisley-Cox, Julia; University of Oxford, Nuffield Department of Primary Care Sciences
**Primary Subject Heading**:	Epidemiology
Secondary Subject Heading:	Infectious diseases
Keywords:	COVID-19, EPIDEMIOLOGY, PUBLIC HEALTH

I, the Submitting Author has the right to grant and does grant on behalf of all authors of the Work (as defined in the below author licence), an exclusive licence and/or a non-exclusive licence for contributions from authors who are: i) UK Crown employees; ii) where BMJ has agreed a CC-BY licence shall apply, and/or iii) in accordance with the terms applicable for US Federal Government officers or employees acting as part of their official duties; on a worldwide, perpetual, irrevocable, royalty-free basis to BMJ Publishing Group Ltd ("BMJ") its licensees and where the relevant Journal is co-owned by BMJ to the co-owners of the Journal, to publish the Work in this journal and any other BMJ products and to exploit all rights, as set out in our licence.

The Submitting Author accepts and understands that any supply made under these terms is made by BMJ to the Submitting Author unless you are acting as an employee on behalf of your employer or a postgraduate student of an affiliated institution which is paying any applicable article publishing charge ("APC") for Open Access articles. Where the Submitting Author wishes to make the Work available on an Open Access basis (and intends to pay the relevant APC), the terms of reuse of such Open Access shall be governed by a Creative Commons licence – details of these licences and which Creative Commons licence will apply to this Work are set out in our licence referred to above.

Other than as permitted in any relevant BMJ Author's Self Archiving Policies, I confirm this Work has not been accepted for publication elsewhere, is not being considered for publication elsewhere and does not duplicate material already published. I confirm all authors consent to publication of this Work and authorise the granting of this licence.

For peer review only

Quantifying the association between ethnicity and COVID-19 mortality: A national cohort study protocol

Hajira Dambha-Miller[1], Pui Tan[2,] Defne Saatci[2], Ashley Clift[2], Francesco Zaccardi[3], Carol Coupland[4], Patrick Locufier[5], Firoza Davies[5], Kamlesh Khunti[3], Simon Griffin[6] and Julia Hippisley-Cox[2]

1. Division of Primary Care and Population Health, University of Southampton
2. Nuffield Department of Primary Care Health Sciences, University of Oxford
3. Leicester Diabetes Centre, Leicester General Hospital, University of Leicester, Leicester, UK
4. Division of Primary Care, School of Medicine, University of Nottingham
5. PPI representative
6. Department of Public Health and Primary Care, University of Cambridge

Correspondence: Dr Hajira Dambha-Miller, Primary Care and Population Health, University of Southampton, Southampton, SO16 5ST, Email: hajiradambha@doctors.org.uk

For peer review only

Abstract

Introduction

Recent evidence suggests that ethnic minority groups are disproportionately at increased risk of hospitalisation and death from SARS-CoV-2 infection. Population-based evidence on potential explanatory factors across minority groups and within sub-groups is lacking. This study aims to quantify the association between ethnicity and the risk of hospitalisation and mortality due to COVID-19.

Methods and analysis

A retrospective cohort study of adults registered across a representative and anonymised national primary care database (QResearch) that includes data on 10 million people in England. Sociodemographic, deprivation, clinical and domicile characteristics will be summarised and compared across ethnic subgroups (categorised as per 2011 census). Cox models will be used to calculate hazard ratios for hospitalisation and COVID-19 mortality associated with ethnic group. Potential confounding and explanatory factors (such as demographic, socioeconomic and clinical) will be adjusted for within regression models. The percentage contribution of distinct risk factor classes to the excess risks seen in ethnic groups/sub-groups will be calculated.

Ethics and dissemination

The study is undergone ethics review in accordance with the QResearch agreement (reference OX102). Findings will be disseminated through peer-reviewed manuscripts, presentations at scientific meetings and conferences with national and international stakeholders.

Strengths and limitations

- The Q-Research database includes a large sample that represents 18% of the English population
- As the data is based on primary care clinical entries, recording of exposures and national linkage to outcomes but this may vary in completeness.
- Total COVID-19 cases in the cohort will be under-estimated as national systematic testing is still being established in the UK

For peer review only - http://bmjopen.bmj.com/site/about/guidelines.xhtml

Introduction

Recent reports and early observational data suggest that the prevalence of COVID-19, hospitalisation and deaths are disproportionately higher amongst ethnic minority populations [1–3]. Potential speculative mechanisms include genetic variations, co-morbidities, cultural or behavioural patterns, occupational factors, social constructs and inequalities. In the UK, people with ethnic minority backgrounds constitute 16% of the population but the Intensive Care National Audit and Research Centre report that over 30% of people admitted to intensive care with COVID-19 were from these backgrounds [4,5]. Furthermore, 64% of 119 NHS staff who died from COVID-19 as of 30th April 2020 were from ethnic minority backgrounds [6]. A rapid review on the subject led by Khunti et al. suggests that there are also variations in mortality within broad ethnic minority groups.[3] Most national and international data on COVID-19 are aggregated and have not reported outcome by ethnic group sufficiently to permit examination of within-group differences.

At the time of submitting this manuscript, a limited number of population-based cohort studies regarding COVID-19 have been undertaken worldwide. In the USA, a study within the largest healthcare system, the Veterans Health Administration, reports excess mortality amongst ethnic minorities.[7] In the UK, the availability of high quality, large (multi-million) databases that have linkage across varying information sources are starting to examine this association further. Large primary care database have been used, for example, to develop risk prediction models for adverse COVID-19 outcomes in the general population , and to undertake large scale epidemiological evaluations of COVID-19 risk factors and drug safety [8]. In the UK, two large cohorts have reported to date on the association between ethnicity and COVID-19 related mortality outcomes: the OpenSAFELY Collaborative of 23 million UK records and the NHS England cohort study of over 61 million people. The OpenSAFELY consortium reported that people with Black or Asian backgrounds have a higher risk of death from COVID-19 compared to people who are White [9]. Data from NHS England also showed a greater mortality risk for Asian (HR 1.3) and Black backgrounds (HR 1.7). Further analysis of this data, carried out by the Office of National Statistics (ONS), showed that the mortality risk in Black men and women was 1.9 times higher than in White individuals. Contradictory to a previous study in a smaller, single-state US health system database,, both studies identify a persisting excess risk of COVID-19 specific mortality after accounting for known risk factors, including age, sex, deprivation and co-morbidities such as obesity, diabetes and cardiovascular disease. The causes of these excess risks are poorly understood, incompletely quantified and may vary within and between ethnic minority groups.

Previous studies have been limited by the lack of consideration of additional potential explanatory variables, such as specific medications, rarer comorbidities [10], social differences such as household structure or vaccination status.[9] A recent population-based study, for example, identified a significant interaction between ethnicity and angiotensin enzyme inhibitors (ACEi)/angiotensin receptor blockers (ARBs) for COVID-19 disease [11]. Several smaller studies have also suggested a role for glucose-lowering medications in the ethnicity-dependent variations observed in COVID-19 outcomes [12,13]. Single-centre studies and case-series are providing additional insights into the potential interaction between ethnicity and haematological disorders, such as sickle cell disease [10], in relation to COVID-19. Population-based studies with adequate number of people from different ethnic groups that consider these wider variables are necessary to evaluate the explanatory factors surrounding this risk. Moreover, earlier studies have been limited, larger cohorts that extend to the wider English population are needed for validation and to allow generalisability of findings. Accordingly, in this study we aim to quantify ethnic group-specific risk of SARS-CoV-2 infection, COVID-19 mortality and COVID-19 hospital admission, and examine the effect of non-modifiable (e.g. age and sex) and explanatory factors on the associations between ethnicity and mortality/hospitalisation risk.

For peer review only - http://bmjopen.bmj.com/site/about/guidelines.xhtml

Methods

Study Design

A retrospective cohort study from electronic GP records linked at the individual level to hospital admissions, SARS-CoV-2 testing and death registry databases.

Data sources

We will utilise the QResearch national primary care database that includes 1205 general practices with 10 million records. The sample represents 18% of the English population. QResearch holds data on a heterogeneous and representative ethnic minority population, with comprehensive information on a range of clinical and socioeconomic factors. Further detail on the population included within the Q-Research database and representative is updated regularly on the database website. [14] Additional databases may be included as these become available.

Study period

We will include a period from the date of the first case of COVID-19 in the UK (24th January 2020) until death, deregistration or the 30th October 2020

Inclusion criteria

All adults aged over 18 years registered with a GP practice during the study period and for at least 12 months prior to inclusion. The GP practice registration needs to be linked to a relevant clinical system (i.e. EMIS).

Primary Outcome

The primary outcome will be a COVID-19 related mortality as recorded in the primary care records and linked to ONS data. Details on the extent of linkage available within the database has been reported previously and can be found in full on the Q-Research website.[15] COVID-19 related mortality will be defined as the presence of U07.1 or U07.2 on death certificates or a death from any cause in a patient with a confirmed positive SARS-CoV-2 test in the immediately preceding 28 days.

Secondary Outcomes

COVID-19 hospitalisation was defined as hospitalisation with confirmed or suspected COVID-19 (as per ICD-10 codes), or new hospitalisation in a patient with a positive SARS-CoV-2 test in the immediately preceding 14 days.

Ethnicity definition

A self-reported ethnic group is recorded in medical records. QResearch has ethnicity records in 80% of all patients. Ethnicity will be considered in two ways; first in the 9 high-level categories: White, Indian, Bangladeshi, Pakistani, other Asian, Black African, Black Caribbean, Chinese, 'Other' and then, if numbers allow, further subdivided into the standard 17 categories used by ONS and include the following; English/Welsh/Scottish/Northern Irish/British, Irish, Gypsy or Irish Traveller, Any other White background, White and Black Caribbean, White and Black African, White and Asian, Any other Mixed/Multiple ethnic backgrounds, Indian, Pakistani, Bangladeshi, Chinese, Any other Asian background, African, Caribbean, Any other Black/African/Caribbean background, Arab or any other ethnic group.

For peer review only - http://bmjopen.bmj.com/site/about/guidelines.xhtml

Explanatory Variables

We will extract data (where available) corresponding to the following potential exposure and confounding variables. This list provided below is indicative and correct as of 29h August 2020, but will be kept under review as new information arises in the literature:

Demographic variables

- Ethnicity (9 categories, and numbers permitting, sub-division into 17 ONS groups)
- Sex
- Age (continuous variable)
- Geographical region

Concurrent medication

- Glucose-lowering medications (metformin, sulfonylureas, thiazolindione, SGLT-2 inhibitors, DPP-4 inhibitors, GLP-1R agonists) and insulin therapy
- Hormone therapy (in women)

Clinical/blood test assessments

- Body mass index
- Blood pressure (systolic and diastolic)
- HbA1c
- Total cholesterol and LDL
- Liver function tests, including alanine aminotransferase
- eGFR

Comorbidities

- Sickle Cell Status [10]
    - Sickle Trait
    - Sickle Cell Disease

- Diabetes (type 1 and type 2 separately) [13,16]
    - Diabetes duration

- Chronic kidney disease
    - CKD stage 3
    - CKD stage 4
    - CKD stage 5 (including those with requiring dialysis or ever receiving a transplant)

- Cancer
    - Active or past haematological or solid cancer

- Transplant (solid organ or bone marrow)
- Vaccination status

Key confounding variables

Demographic variables

- Quintile of Townsend deprivation score. This is an area-level quintile score based on the patients' postcode. Originally developed by Townsend, it includes unemployment (as a percentage of those aged 16 and over who are economically active); non-car ownership (as a percentage of all households); non-home ownership (as a percentage of all households) and household overcrowding. These variables are measured for a given area of approximately 120 households, via the 2011 census, and combined to give a "Townsend score" for that area. A higher Townsend score implies a greater level of deprivation.

For peer review only - http://bmjopen.bmj.com/site/about/guidelines.xhtml

- Residence type
  - Lives in a care home (nursing home or residential care)
  - Homelessness
  - Lives in own home
- Household characteristics
  - Age of household members
  - Number of household inhabitants

Lifestyle factors

- Smoking status coded as non-smoker, ex-smoker, light [<10 cigarettes/day], moderate [10-19], heavy [20+])

Comorbidities

- Cardiovascular disease
- Hypertension
- Chronic respiratory disease
- Liver cirrhosis
- Depression
- Dementia
- Other chronic neurological diseases
- Severe mental health illness
- Learning Disability

Concurrent medication

- Lipid-lowering medications
- Antihypertensive medications

All predictor variables will be based on the latest coded information recorded in the GP record prior to entry to the cohort.

Sample size calculation

As of the 9th Aug 2020, there had been 41,686 COVID-19 deaths in England out of a total population of 56 million. Assuming a 0.074% mortality rate in the general population, to detect a conservative estimate of a hazard ratio of 1.2 for COVID-19 mortality in the ethnic minority population that represents 18.6% of the study population (based on recent QResearch studies) with 90% power and a significance level of 0.01, we would need a sample size of 3,995,350 individuals with 2,957 COVID-19 deaths. As recent studies of COVID-19 using QResearch have included cohorts of over 8 million adults with over 5,000 COVID-19 deaths, even with 40% missing data, our study is sufficiently powered.

Statistical methods

Participant characteristics will be summarised by ethnicity, age, sex, co-morbidities and outcome with appropriate summary statistics. We will describe SARS-CoV-2 infection, COVID-19 hospitalisation and COVID-19 mortality rates in the general population and stratify this by ethnicity. Plots of survival curves stratified by ethnic group will be generated using the Kaplan-Meier method and compared with the log-rank test. We will use Cox proportional hazards models to estimate the unadjusted and adjusted hazard ratios for associations between ethnicity and the outcomes of interest. When proportional hazards assumptions are not met, we will consider using more flexible models which do not assume proportionality of hazards e.g. flexible parametric survival models. Subsequently, we will seek to quantify the contribution of risk factor groups to any observed increased mortality or hospitalisation risks in people of non-white ethnicity. For all analyses, we will conduct complete case analyses, and then separately impute for missing values e.g. ethnic group, BMI, deprivation quintile and smoking status using five imputations incorporating all model outcomes and predictors with model coefficients and standard errors pooled in accordance with Rubin's rules. Data will be analysed using STATA

For peer review only - http://bmjopen.bmj.com/site/about/guidelines.xhtml

(version 16). Our study will be conducted and findings reported in line with the STROBE [17] and RECORD guidelines for observational studies using routinely collected health data[18].

Patient and public involvement

Patient representatives have been involved in the development of the study aims and design. We sought feedback from study inception and design from patients and the public within the Centre for BME Health, Leicester. We additionally have two public representatives as part of the study team whose contributions have been acknowledged in the authorship list of this manuscript. They will continue to be involved in all stages of study with additional public and patient views sought for dissemination strategies.

Ethics and dissemination

The project has been independently peer-reviewed and received ethics approval by the QResearch Scientific board (reference OX102). Findings will be disseminated through peer-reviewed manuscripts, presentations at scientific meetings and conferences with national and international stakeholders.

Data sharing agreement

Data are available by direct requests to the Q-Research database. We are working towards making our code publicly available.

Discussion

Our findings aim to provide rapid evidence on patterns of COVID-19 and associated mortality across and within ethnic groups in England. This study will be one of the largest COVID-19 cohorts, with a representative population across both rural and urban areas. Our cohort size will enable us to detect national-level variations within ethnic minority subgroups across a large geographical area. Thus, to some extent, overcome selection bias limitations of previous cohort studies. Our findings have the potential to inform targeted mitigation public health strategies and could alter clinical thresholds for at-risk patients presenting with the infection.

As we are using routinely recorded clinical data from primary care records rather than prospectively collecting the data, we anticipate that our study will be subject to limitations. First, the precision and completeness of routine NHS patient records will vary although previous work from QResearch suggests that key variables have high levels of accuracy and completeness. Second, our sample is likely to under-estimate the total number of people in the population with COVID-19 due to the lack of systematic national testing programmes which are still being rolled out, and also due to false-negative test results. There is also some evidence that testing rates are lower amongst ethnic minority groups. Our confirmed SARS-CoV-2 infection cases will additionally over-represent people with more severe disease who died or were hospitalised. During the earlier part of the pandemic, testing in the UK was limited to the hospital setting and people with COVID-19 who were not admitted or recovered (who may be from ethnic minority groups) are less likely to be captured in the data. Further, the admission criteria for ITU varied during the course of the pandemic with particular characteristics such as older age resulting in a lower chance of admission at a time when high admission rates were anticipated, which may introduce a selection bias.

Funding

For peer review only - http://bmjopen.bmj.com/site/about/guidelines.xhtml

This work is funded by an MRC grant (MR/V027778/1). SJG is supported by an MRC Epidemiology Unit programme: MC_UU_12015/4. The University of Cambridge has received salary support in respect of SJG from the NHS in the East of England through the Clinical Academic Reserve. HDM is a National Institute for Health Research funded Academic Clinical Lecturer. JHC receives support from the NHS and National Institute for Health Research and various research councils. KK and FZ are supported by the National Institute for Health Research (NIHR) Applied Research Collaboration East Midlands (ARC EM) and the NIHR Leicester Biomedical Research Centre (BRC). The views and opinions expressed by authors in this publication are those of the authors and do not necessarily reflect those of the UK National Institute for Health Research (NIHR) or the Department of Health and Social Care.

Competing Interests

JHC is founder and director of QResearch database which is not-for-profit organisation with EMIS (leading commercial supplier of IT for 55% of general practices in the UK). JHC is co-owner of ClinRisk Ltd and was a paid director until June 2019. ClinRisk Ltd develops open and closed source software to ensure the reliable and updatable implementation of clinical risk equations within clinical computer systems to help improve patient care outside the submitted work. KK is national lead for NIHR ARC ethnicity and diversity, Director for the University of Leicester Centre for BME Health, Trustee of the South Asian Health Foundation and member of the Independent SAGE. The authors declare that no support from any organisation and no financial relationships have influenced the submitted work.

Contributor statement

HDM led the study conceptualisation, wrote the first draft and revised the protocol, PT, DS and AC critically revised the manuscript. FZ critically revised the manuscript and contributed to the statistical methods, CC critically revised the manuscript and contributed to the statistical methods, PL and FD critically revised the manuscript as a PPI contributors, KK, SG and JHC contributed to study conceptualisation, study design and revised the protocol. The corresponding author attests that all listed authors meet authorship criteria and that no others meeting the criteria have been omitted. HDM is the guarantor.

References

1 Pareek M, Bangash M, Pareek N et al. 2020. Ethnicity and COVID-19: an urgent public health research priority - The Lancet. doi:OI:https://doi.org/10.1016/S0140-6736(20)30922-3
2 Laurencin CT, McClinton A. The COVID-19 Pandemic: a Call to Action to Identify and Address Racial and Ethnic Disparities. *Journal of Racial and Ethnic Health Disparities* 2020;:1. doi:10.1007/s40615-020-00756-0
3 BAME COVID-19 DEATHS - What do we know? Rapid Data & Evidence Review - CEBM. https://www.cebm.net/covid-19/bame-covid-19-deaths-what-do-we-know-rapid-data-evidence-review/ (accessed 6 May 2020).
4 ICNARC – Reports. https://www.icnarc.org/Our-Audit/Audits/Cmp/Reports (accessed 4 May 2020).
5 Ethnic minority deaths and Covid-19, The King's Fund. https://www.kingsfund.org.uk/blog/2020/04/ethnic-minority-deaths-covid-19 (accessed 4 May 2020).
6 Rimmer A. Covid-19: Two thirds of healthcare workers who have died were from ethnic minorities. *BMJ* 2020;**369**:m1621. doi:10.1136/bmj.m1621
7 Rentsch ID CT, Kidwai-Khan F, Tate JP, *et al.* Patterns of COVID-19 testing and mortality by race and ethnicity among United States veterans: A nationwide cohort study. doi:10.1371/journal.pmed.1003379
8 Clift AK, Coupland CAC, Keogh RH, *et al.* Living risk prediction algorithm (QCOVID) for risk of hospital admission and mortality from coronavirus 19 in adults: national derivation and validation cohort study. *The BMJ* 2020;**371**. doi:10.1136/bmj.m3731
9 OpenSAFELY Collaborative T, Mathur R, Rentsch CT, *et al.* Ethnic differences in COVID-19 infection, hospitalisation, and mortality: an OpenSAFELY analysis of 17 million adults in

For peer review only - http://bmjopen.bmj.com/site/about/guidelines.xhtml

England. *medRxiv* 2020;:2020.09.22.20198754. doi:10.1101/2020.09.22.20198754
10 Arlet JB, de Luna G, Khimoud D, *et al.* Prognosis of patients with sickle cell disease and COVID-19: a French experience. The Lancet Haematology. 2020;**7**:e632–4. doi:10.1016/S2352-3026(20)30204-0
11 Hippisley-Cox J, Young D, Coupland C, *et al.* Risk of severe COVID-19 disease with ACE inhibitors and angiotensin receptor blockers: cohort study including 8.3 million people. *Heart (British Cardiac Society)* 2020;**106**:1503–11. doi:10.1136/heartjnl-2020-317393
12 Zhu L, She ZG, Cheng X, *et al.* Association of Blood Glucose Control and Outcomes in Patients with COVID-19 and Pre-existing Type 2 Diabetes. *Cell Metabolism* 2020;**31**:1068-1077.e3. doi:10.1016/j.cmet.2020.04.021
13 Scheen AJ, Marre M, Thivolet C. Prognostic factors in patients with diabetes hospitalized for COVID-19: Findings from the CORONADO study and other recent reports. Diabetes and Metabolism. 2020;**46**:265–71. doi:10.1016/j.diabet.2020.05.008
14 Summary of GP population characteristics - QResearch. https://www.qresearch.org/data/summary-of-gp-population-characteristics/#h-H2_0 (accessed 24 Jan 2021).
15 Summary Analysis of Linked Mortality Data - QResearch. https://www.qresearch.org/data/summary-analysis-of-linked-mortality-data/ (accessed 24 Jan 2021).
16 Barron E, Bakhai C, Kar P, *et al.* Associations of type 1 and type 2 diabetes with COVID-19-related mortality in England: a whole-population study. *The Lancet Diabetes and Endocrinology* 2020;**8**:813–22. doi:10.1016/S2213-8587(20)30272-2
17 Von Elm E, Altman DG, Egger M, *et al.* The Strengthening the Reporting of Observational Studies in Epidemiology (STROBE) statement: Guidelines for reporting observational studies. Annals of Internal Medicine. 2007;**147**:573–7. doi:10.7326/0003-4819-147-8-200710160-00010
18 Benchimol EI, Smeeth L, Guttmann A, *et al.* The REporting of studies Conducted using Observational Routinely-collected health Data (RECORD) Statement. *PLoS Medicine* 2015;**12**. doi:10.1371/journal.pmed.1001885

For peer review only

For peer review only - http://bmjopen.bmj.com/site/about/guidelines.xhtml

STROBE 2007 (v4) checklist of items to be included in reports of observational studies in epidemiology*

Checklist for cohort, case-control, and cross-sectional studies (combined)

Section/Topic	Item #	Recommendation	Reported on page #
**Title and abstract**	1	(*a*) Indicate the study's design with a commonly used term in the title or the abstract	2-3
		(*b*) Provide in the abstract an informative and balanced summary of what was done and what was found	2
**Introduction**			
Background/rationale	2	Explain the scientific background and rationale for the investigation being reported	3
Objectives	3	State specific objectives, including any pre-specified hypotheses	3
**Methods**			
Study design	4	Present key elements of study design early in the paper	4
Setting	5	Describe the setting, locations, and relevant dates, including periods of recruitment, exposure, follow-up, and data collection	4-5
Participants	6	(*a*) *Cohort study*—Give the eligibility criteria, and the sources and methods of selection of participants. Describe methods of follow-up<br>*Case-control study*—Give the eligibility criteria, and the sources and methods of case ascertainment and control selection. Give the rationale for the choice of cases and controls<br>*Cross-sectional study*—Give the eligibility criteria, and the sources and methods of selection of participants	5-6
		(*b*) *Cohort study*—For matched studies, give matching criteria and number of exposed and unexposed<br>*Case-control study*—For matched studies, give matching criteria and the number of controls per case	n/a
Variables	7	Clearly define all outcomes, exposures, predictors, potential confounders, and effect modifiers. Give diagnostic criteria, if applicable	6-7
Data sources/ measurement	8*	For each variable of interest, give sources of data and details of methods of assessment (measurement). Describe comparability of assessment methods if there is more than one group	7
Bias	9	Describe any efforts to address potential sources of bias	7
Study size	10	Explain how the study size was arrived at	6
Quantitative variables	11	Explain how quantitative variables were handled in the analyses. If applicable, describe which groupings were chosen and why	6
Statistical methods	12	(*a*) Describe all statistical methods, including those used to control for confounding	6
		(*b*) Describe any methods used to examine subgroups and interactions	n/a
		(*c*) Explain how missing data were addressed	n/a
		(*d*) *Cohort study*—If applicable, explain how loss to follow-up was addressed<br>*Case-control study*—If applicable, explain how matching of cases and controls was addressed	n/a

For peer review only

		Cross-sectional study—If applicable, describe analytical methods taking account of sampling strategy	
		(*e*) Describe any sensitivity analyses	8
**Results**			
Participants	13*	(a) Report numbers of individuals at each stage of study—eg numbers potentially eligible, examined for eligibility, confirmed eligible, included in the study, completing follow-up, and analysed	8-9
		(b) Give reasons for non-participation at each stage	n/a
		(c) Consider use of a flow diagram	n/a
Descriptive data	14*	(a) Give characteristics of study participants (eg demographic, clinical, social) and information on exposures and potential confounders	8-9
		(b) Indicate number of participants with missing data for each variable of interest	n/a
		(c) *Cohort study*—Summarise follow-up time (eg, average and total amount)	n/a
Outcome data	15*	*Cohort study*—Report numbers of outcome events or summary measures over time	n/a
		Case-control study—Report numbers in each exposure category, or summary measures of exposure	n/a
		Cross-sectional study—Report numbers of outcome events or summary measures	n/a
Main results	16	(*a*) Give unadjusted estimates and, if applicable, confounder-adjusted estimates and their precision (eg, 95% confidence interval). Make clear which confounders were adjusted for and why they were included	n/a
		(*b*) Report category boundaries when continuous variables were categorized	n/a
		(*c*) If relevant, consider translating estimates of relative risk into absolute risk for a meaningful time period	n/a
Other analyses	17	Report other analyses done—eg analyses of subgroups and interactions, and sensitivity analyses	7
**Discussion**			
Key results	18	Summarise key results with reference to study objectives	n/a
Limitations	19	Discuss limitations of the study, taking into account sources of potential bias or imprecision. Discuss both direction and magnitude of any potential bias	7
Interpretation	20	Give a cautious overall interpretation of results considering objectives, limitations, multiplicity of analyses, results from similar studies, and other relevant evidence	n/a protocol study
Generalisability	21	Discuss the generalisability (external validity) of the study results	7
**Other information**			
Funding	22	Give the source of funding and the role of the funders for the present study and, if applicable, for the original study on which the present article is based	7

*Give information separately for cases and controls in case-control studies and, if applicable, for exposed and unexposed groups in cohort and cross-sectional studies.

Note: An Explanation and Elaboration article discusses each checklist item and gives methodological background and published examples of transparent reporting. The STROBE checklist is best used in conjunction with this article (freely available on the Web sites of PLoS Medicine at http://www.plosmedicine.org/, Annals of Internal Medicine at http://www.annals.org/, and Epidemiology at http://www.epidem.com/). Information on the STROBE Initiative is available at www.strobe-statement.org.

For peer review only
